# Transplacental transferred anti-SARS-CoV-2 neutralizing antibodies in unvaccinated pregnant women in Cameroon occurred during the COVID-19 pandemic, but not in the pre-pandemic period

**DOI:** 10.3389/fimmu.2025.1628102

**Published:** 2025-11-19

**Authors:** Reine Medouen Ndeumou Seumko’o, Thandeka Moyo-Gwete, Tandile Hermanus, Sosthene Hillary Matabou Tene, Romeo Brice Dieffouo Djounda, Chris Marco Mbianda Nana, Bernard Marie Zambo Bitye, Tekougang Berenice Kenfack Zangue, Bodin Darcisse Kwanou Tchakounte, Eitel Mpoudi Ngolle, Diane Wallace Taylor, Rose Gana Fomban Leke, Rosette Megnekou, Penny L. Moore, Livo Forgu Esemu

**Affiliations:** 1 Department of Animals Biology and Physiology, University of Yaoundé I, Yaounde, Cameroon; 2 The Biotechnology Center, University of Yaoundé I, Yaoundé, Cameroon; 3 Institute of Medical Research and Medicinal Plant Studies, Centre for Research on Emerging and Reemerging Diseases, Yaounde, Cameroon; 4 Antibody Immunity Research Unit, School of Pathology, Faculty of Health Sciences, University of the Witwatersrand, Johannesburg, South Africa; 5 Centre for HIV and STIs, National Institute for Communicable Diseases of the National Health Laboratory Service, Johannesburg, South Africa; 6 Department of Biochemistry, University of Yaoundé I, Yaoundé, Cameroon; 7 Epidemiology and Public Health Department, Centre Pasteur du Cameroun, Yaounde, Cameroon; 8 Department of Tropical Medicine, Medical Microbiology and Pharmacology, University of Hawaii at Manoa, John A. Burns School of Medicine, Honolulu, HI, United States; 9 Centre for the AIDS Program of Research in South Africa (CAPRISA), Durban, South Africa; 10 Infectious Diseases and Oncology Research Institute (IDORI), Faculty of Health Sciences, University of the Witwatersrand, Johannesburg, South Africa; 11 Department of Biomedical Sciences, University of Buea, Buea, Cameroon

**Keywords:** COVID-19, SARS-CoV-2, pregnancy, neutralizing antibodies, transplacental antibody transfer

## Abstract

**Introduction:**

Neutralizing antibodies (NAbs) are critical for protection against SARS-CoV-2, but there is limited information on their role in pregnancy, especially among Cameroonian women. Here, we aimed to determine the prevalence of pan-coronavirus reactive antibodies from pregnant women sampled before and during the COVID-19 pandemic.

**Methods:**

Plasma samples from 629 women in the second trimester and 661 at delivery were collected pre-COVID-19 and from 39 women at delivery during COVID-19 in Yaoundé, Cameroon. All samples were screened using the Abbott Panbio™ COVID-19 rapid diagnostic test (RDT). Enzyme-linked immunosorbent assays (ELISA) and the spike-pseudotyped lentivirus neutralization assay were done to measure antibody binding and neutralizing capacity in 118 and 33 samples, respectively.

**Results:**

Before the pandemic, 16.5% (213/1290) of pregnant women were seropositive for cross-reactive anti-SARS-CoV-2 antibodies by RDT, while 12.2% (11/90) were seropositive to antibody binding by ELISA. Additionally, no correlation was found between cross-reactivity against the spike protein of SARS-CoV-2 and the HCoVs-OC43 and HcoVs-NL63 spikes. However, during the pandemic, 53.8% (21/39) of women sampled at delivery were seropositive by RDT, all women (28/28-100%) were seropositive by ELISA and 90% (20/22) of the samples from pregnant women tested for neutralization (20/22) had detectable neutralizing antibody responses during the COVID-19 pandemic. A transplacental transfer of binding antibodies from the mother to the child was found in 76.9% (30/39) of the tested dyads with a high prevalence during pandemic (26/28-86.7%) than prior the pandemic (4/11-13.3%).

**Discussion:**

This study goes to reinforce the need for vaccination as though, all participants elicited a response towards endemic coronaviruses before the COVID-19 pandemic, a very small fraction of participants had binding antibodies which cross-react with SARS-CoV-2 and none of these were neutralizing. Anti-SARS-CoV-2 antibodies from the studied pregnant Cameroonian women at delivery during the pandemic had neutralizing activity against the founder variant and were efficiently transferred to the newborn. However the neutralization against other variants of concern warrants future investigation.

## Introduction

Coronaviruses (CoVs) are large, single-stranded RNA viruses that belong to the order Nidovirales and family Coronaviridae. Based on variations in protein sequences, CoVs can be divided into four phylogenetic groups (or genera): alpha and beta (which are known to infect mammals) and delta and gamma (which are known to infect both mammals and birds) (1, 2). The COVID-19 causal agent, SARS-CoV-2, was initially discovered in Wuhan, China, in December 2019 (3). SARS-CoV-2 was rapidly transmitted globally, disrupting structures and systems, and causing millions of deaths. In Africa, Sub-Saharan Africa (SSA) was expected to experience a high burden of incidence, hospitalizations, and deaths based on epidemiological modeling (4). Nigeria reported the first case of COVID-19 in SSA on January 28, 2020 (5), providing and important epidemiological context for interpreting the timing and relevance of our serological findings. Cameroon confirmed its first case on March 6, 2020 (6). By December 22, 2024, the country had reported a total of 125,279 cases and 1,974 deaths. However, Africa is reported to be among the least affected regions by the pandemic and many hypotheses have been suggested: (i) the young population in Sub-Saharan Africa; (ii) SARS-CoV-2 persistence and spread disadvantaged by climatic and environmental factors; (iii) social distancing favored by the lifestyle in rural/less developed areas, which limits the spread of the disease; (iv) underestimation of morbidity and mortality counts due to poor testing coverage, reflecting weak health systems; (v) a rapid activation of the natural innate non-specific immunity due to an overexposure to pathogens; and (vi) specific immune response following a previous contact with viruses sharing common antigenic profiles with SARS-CoV-2 (7, 8).

The potential low circulation of SARS-CoV-2 in Africa may be explained by the existence of specific pre-pandemic antibodies, responsible for cross-immunity during the pandemic (9). Among all existing CoVs, seven are infectious to humans among which four are endemic coronaviruses (HCoV-229E, HCoV-NL63, HCoV-OC43, and HCoVHKU1) are low-pathogenic human coronaviruses that typically only cause mild upper respiratory tract infections (10). The others three, Middle East Respiratory Syndrome Coronavirus (MERS-CoV) (11) and the severe acute respiratory syndrome coronaviruses (SARS-CoV1 and SARS-CoV-2) (12–14) are highly pathogenic human coronaviruses that have been found to cause severe acute respiratory diseases. Prior exposure to other coronaviruses might provide some degree of humoral cross-protection against SARS-CoV-2 infection, which would lower the incidence and/or severity of COVID-19 infections.

Pregnant women are a highly vulnerable population, as the robustness of their immune systems declines. Pregnancy alters immunological homeostasis as well as balance in the circulatory and respiratory systems (15). Pregnant women infected with SARS-CoV-2 are more likely than non-infected women to experience severe disease, including a higher risk of death, invasive ventilation, and intensive care unit admission (16, 17). Nwosu and collaborators showed in their study in 2021 that, among 971 participants, the seroprevalence of anti-SARS-CoV-2 IgG antibodies was 29.2% which was about 322 times greater than the 0.09% nationwide attack rate implied by COVID-19 case counts at the time (18). However, there is a paucity in serological data in pregnant women in understanding immunity to SARS-CoV-2 in this vulnerable group. We hypothesized that the existence of pre-existing immunity against other circulating human coronaviruses confers protection to SARS-CoV-2 in pregnant women sampled during the pre-COVID-19 era. So, we conducted this study to determine the prevalence of pan-coronavirus antibodies from pregnant women sampled before the COVID-19 pandemic in Yaounde, the neutralizing capacity of these cross-reactive anti-SARS-CoV-2 antibodies in those seropositive samples and provide evidence of the transplacental transfer of antibodies from mothers to their newborn babies.

## Methodology

### Study design

We conducted a retrospective cross-sectional study firstly on archived plasma samples collected between 2009 and 2018 before the COVID-19 pandemic in Cameroon and secondly on samples collected during the COVID-19 pandemic period between 2021 and 2022.

### Ethics approval and consent to participate

This study utilized de-identified, anonymized plasma samples biobanked after malaria research studies (19, 20), and it was approved by the Centre Regional Ethics Committee for Human Health Research (CE-N^0^0146/CRERSHC/2021). Administrative authorizations were obtained from the Ministry of Public Health of Cameroon and from local health centers. For samples collected during the pandemic period (2021–2022), authorization was granted by the directors of the three selected health facilities. An informational note was provided to all eligible participants, who then gave their written informed consent prior to enrollment in the study. The confidentiality of study participants was maintained using identification codes.

### Study site

Samples prior the outbreak of COVID-19 in Cameroon used in this study were collected in three geographic areas of the city of Yaoundé in Cameroon, a rural area in Ngali, a peri-urban area at the Marie-Reine d’Etoudi Medical Center and an urban area at the Yaoundé Central Hospital and the Efoulan District Hospital. Processing was carried out at the Immunology Laboratory of the Biotechnology Center of the University of Yaoundé I and at the Center for Research on Emerging and Reemerging Diseases (CREMER) of the Institute of Medical Research and Medicinal Plant Studies.

### Sampling and eligibility criteria

**Figure 1:**
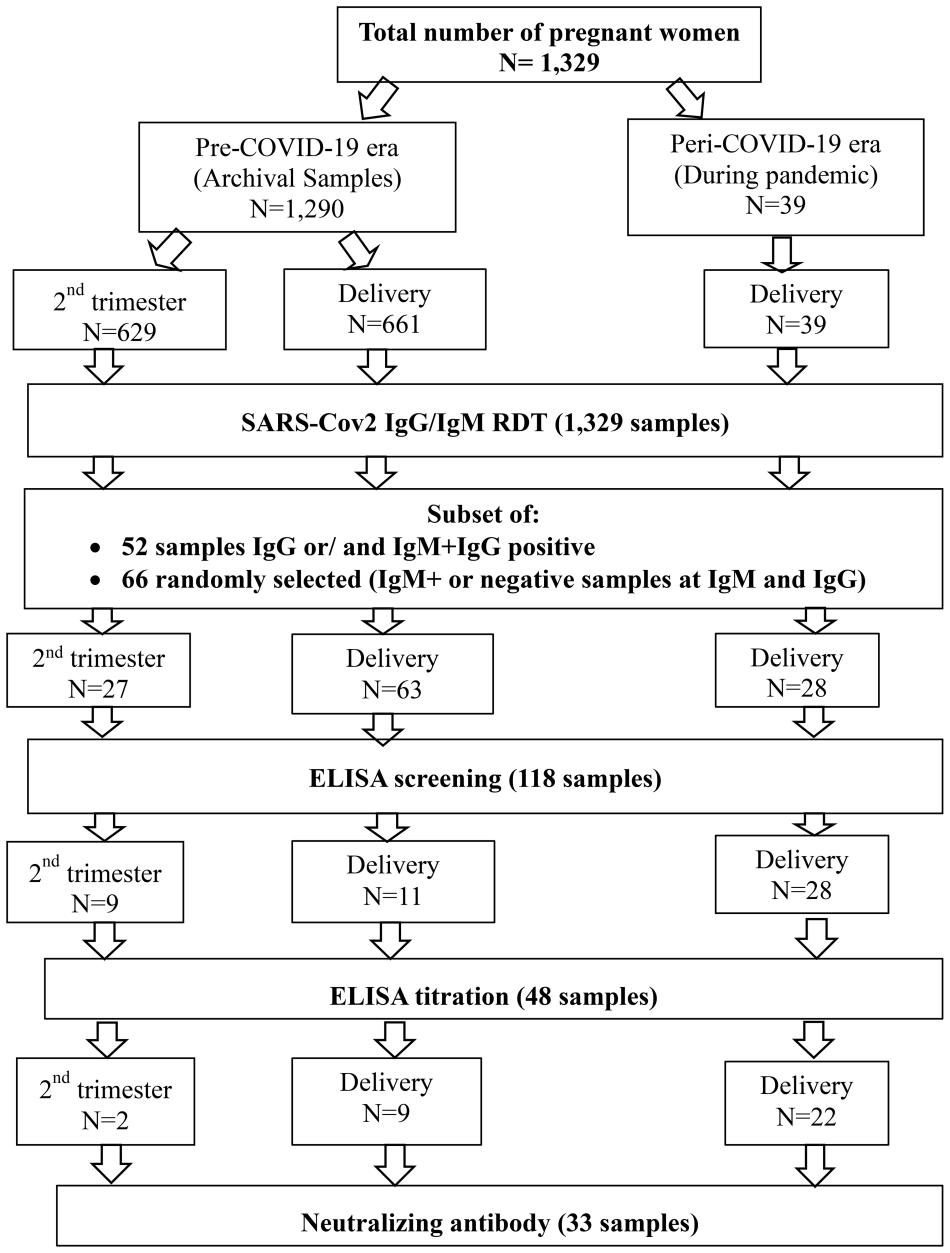
Sample flow chart. A total of 1,329 samples were collected from pregnant women during the pre-COVID-19 and peri-COVID-19 periods. Samples were obtained at two time points: during the second trimester and at delivery. All samples underwent screening with a rapid diagnostic test (RDT). Following this, antibody binding was measured using ELISA. For those who were seropositive, neutralizing antibodies were assessed.

Overall, we included 1,329 plasma samples from pregnant women among which 1,290 were archival samples collected before the COVID-19 pandemic (pre-COVID-19) and 39 plasma samples from women at delivery collected during the pandemic (peri-COVID-19). Those samples were previously anonymized and codified as per institutional biobanking procedures. The archival samples were collected from 629 consenting pregnant women recruited during the second trimester between the 12^th^ and 27^th^ weeks of pregnancy and 661 at delivery. All women were aged between 15 to 45 years (**Figure 1**).

### Clinical and laboratory procedures

SARS-CoV-2 antibodies were tested using the Abbott Panbio™ COVID-19 IgG/IgM Rapid Diagnostic Test as per manufacturer’s instructions. Assay performance as reported by the manufacturer was as follows: sensitivity (97.8%); specificity (92.8%); precision (>99% both for intra-assay and inter-assay assessments) (21). Briefly, plasma samples were mixed by low-speed vortex after which 10µL of supernatant was applied to the specimen well (S) of the test device. Two drops (approximately 60µL) of buffer were added and incubated for 10 minutes before reading the test device.

### SARS-CoV-2 and HCoV antigen synthesis

For serology assays, recombinant SARS-CoV-2 Hexapro spike protein was used for this study (22). The SARS-CoV-2, OC43 and NL63 spike proteins were expressed in Human Embryonic Kidney (HEK) 293F suspension cells by transfecting the cells with the spike plasmid as previously described (23). After 6 days, proteins were purified using a nickel resin followed by size-exclusion chromatography. Relevant fractions were collected and frozen at -80°C until use.

### Anti-SARS-CoV-2 antibodies detection

Participants’ serostatus was assessed using an Enzyme-Linked Immunosorbent Assay (ELISA) for quantitative detection of anti-SARS-CoV-2 Spike IgG in plasma samples (24, 25). 96-well high-binding plates were coated separately with 50 µL of SARS-CoV-2, OC43 or NL63 spike protein diluted in 2 µg/ml of 1x PBS and incubated overnight at 4°C. After the overnight incubation of the plates coated by the antigens, the plates were washed 3 times with 250 μL of wash buffer per well using an automated plate washer. 5% skimmed milk powder diluted in wash buffer (1x PBS, 0.05% Tween 20 and distilled water) was used as blocking buffer. 200 µL of blocking buffer was then added to each well and incubate at room temperature (RT) for one hour. After this incubation, the plates were washed as previously described and 100 µL plasma samples pre-diluted to a 1:100 dilution in blocking buffer, were added to the plates and incubated for 75 minutes. Titrations were performed by doing a serial dilution of each positive sample in blocking buffer to determine the effective concentration of the antibodies present. Following incubation, plates were washed and the secondary antibody diluted at 1:3000 in blocking buffer was added to the plates and further incubated for 60 minutes then washed. 100 µL TMB substrate (*Thermofisher Scientific*) was then added to all the wells and incubated for 5 min at RT in the dark. Upon stopping the reaction with 1M H_2_SO_4_, absorbance was measured at a 450 nm wavelength and used to calculate the half maximal effective concentration (EC50) using *GraphPad Prism v9.0.0.121.* The mAb CR3022 was used as positive control and Palivizumab was used as a negative control. A Pregnant women was considered to be seropositive at anti-SARS-CoV-2 IgG antibodies when OD450nm ≥ 0.4.

### Spike plasmid, lentiviral pseudovirus production and neutralization assay

Pseudoviruses were produced by co-transfection of HEK293T cells with the SARS-CoV-2 614G spike (D614G) plasmids in conjunction with a firefly luciferase encoding lentivirus backbone plasmid (pNL4plasmid) with PEIMAX (Polysciences). Culture supernatants were clarified of cells by a 0.45 μM filter and stored at −70°C (26, 27).

The 293T/ACE2.MF cells modified to overexpress human ACE2 were kindly provided by M. Farzan (Scripps Research). Cells were cultured in Dulbecco’s Modified Eagle Medium (DMEM) (Gibco, Life Technologies) containing 10% heat-inactivated serum (FBS) and 3 μg/mL puromycin at 37°C, 5% CO2. Plasma samples were heat-inactivated and clarified by centrifugation. Pseudovirus and serially diluted plasma were incubated for 1 h at 37°C, 5% CO_2_. Cells were added at 1 × 10^4^ cells per well and after 72 h of incubation at 37°C, 5% CO_2_ luminescence was measured as a reduction in luciferase gene expression after single-round infection of 293T/ACE2.MF cells with spike-pseudotyped viruses (26–28).

The threshold value used to determine the neutralization titer was given by the ID50, which represents the dilution at which 50% of viral activity is inhibited. Our starting dilution factor was 25. Then, for all pregnant women with an ID50 greater than 25 were considered positive and their antibody titers were taken into account.

### Statistical analysis

Data collected were entered on *Microsoft Excel 2013 and* Graphpad Prism version 9.0.0.121 was used for statistical analysis. Association analyses were performed using Pearson ChiSquare Test. We used a linear regression in order to understand the relationship between anti-D614G and anti-OC43 and anti-NL63 antibodies. The Mann-Whitney test was also used in this study to determine whether there is a significant difference between the median hemoglobin level of women at the second trimester and for those of women at delivery. The paired t-test was used to compare the means of mother’s anti-SARS-CoV-2 antibodies profile against their newborn’s anti-SARS-CoV-2 antibodies profile. Statistical significance was considered at p<0.05.

## Results

### Characteristics of the study population

In total, 1,329 samples were included in this study, among which 1,290 were archived plasmas while 78 were collected during the COVID-19 period. The socio-demographic parameters are summarized in **Table 1**.

**Table 1:** Characteristics of the study population.

Parameters	N	%	P value
All women	1,329	/	
2^nd^ trimester	629	47.3	**/**
Delivery	700	52.7	
Maternal age, years old, Mean [min-max]	**26.1 [15-44]**	/	**/**
Gravidity			
Primigravidae	199	27.1	<0.0001
Multigravidae	536	72.9	
Parity			
Primiparous	392	35.9	<0.0001
Multiparous	700	64.1	
Area of residence			
Rural area	**126**	**9.5**	/
2^nd^ trimester	103	81.7	
Delivery	26	2.1	
Peri-urban area	**708**	**53.3**	/
2^nd^ trimester	222	31.3	
Delivery	486	68.6	
Urban area	**456**	**34.3**	/
2^nd^ trimester	304	66.7	
Delivery	152	33.3	
Period of collect			/
Pre-COVID-19	**1,290**	**97**	
2^nd^ trimester	629	48.8	
Delivery	661	51.2	
Peri-COVID-19	**39**	**2.9**	
2^nd^ trimester	0	0	
Delivery	39	100	
HGB level, g/dL (mean ± SD)	**11.5 ± 1.7**	/	<0.0001
2^nd^ trimester	11.2 ± 1.6	/	
Delivery	11.9 ± 1.8	/	

Min, minimum; max, maximum; HGB, haemoglobin.

P values bolded means p<0.05 i.e statistically significant.

This study included a total of 1,329 women with 629 (47.3%) in their second trimester and 700 (52.7%) at delivery. The participants’ average age was 26.1 years, with ages ranging from 15 to 44. There was a statistical difference in both gravidity and parity among this study population (p < 0.0001). Among the women in their second trimester, 103 (81.7%) resided in rural areas, and 222 (31.3%) lived in peri-urban areas, while 304 (66.7%) were from urban areas. Among those at delivery, 26 (2.1%) resided in rural areas, 486 (68.6%) lived in peri-urban areas, and 152 (33.3%) were from urban areas. However, there was no statistical variation (p=0.2) of age among women between those areas of residence. Samples were collected during two periods: pre-COVID-19 and peri-COVID-19. The pregnant women who gave birth during the COVID-19 pandemic were significantly (p=0.01) older (average of 28 years old) than those before the COVID-19 pandemic (average of 26 years old). During the pre-COVID-19 period, 629 women (48.8%) were sampled in their second trimester, and 661 women (51.2%) were sampled at delivery. During the peri-COVID-19 period, 39 women were sampled, all at delivery. A significant difference in hemoglobin (HGB) levels was observed between women in their second trimester and those at delivery (p < 0.0001) (**Table 1**).

### Cross-reactive binding antibodies to SARS-CoV-2 in samples collected prior to the COVID-19 pandemic

**Figure 2:**
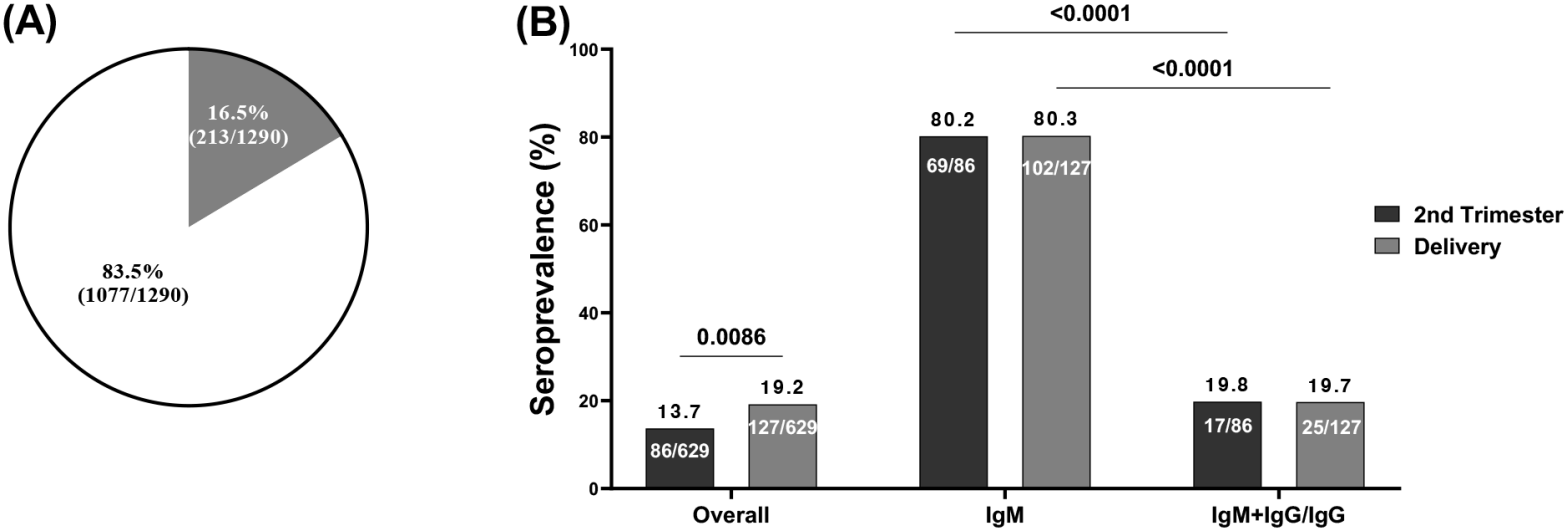
Seroprevalence of cross-reactive anti-SARS-CoV-2 antibodies by RDT. **(A)** Blinded plasma samples from 1290 pregnant women before the pandemic were screened by RDT which showed 213 seropositive pregnant women; **(B)** Among 213 seropositive pregnant women collected during the 2 time points: 86 at the second trimester among which 69 were seropositive for IgM and 17 were seropositive for both IgM and IgG or IgG; and 127 at delivery among which 102 were seropositive for IgM and 25 seropositive for both IgM and IgG or IgG. IgM, Immunoglobulin M; IgG, Immunoglobulin G.

The seroprevalence of pregnant women with cross-reactive anti-SARS-CoV-2 antibodies was 16.5% among 1,290 women tested using RDT (**Figure 2A**) with significantly higher prevalence (p=0.0086) at delivery (19.2%) compared to the prevalence obtained during the second trimester (13.7%) (**Figure 2A**). Since this study primarily focuses on seroprotection, which is mainly represented by neutralization, we laid emphasis on samples with IgG which is efficiently transported across the placenta to the fetus. A higher positivity rate (p<0.0001) to IgM (80.3%) compared to IgM/IgG (19.7%) was seen among the pregnant women during the second trimester (80.2% and 19.8% respectively) and at delivery (80.3% and 19.7% respectively) as shown (**Figure 2B**).

**Figure 3:**
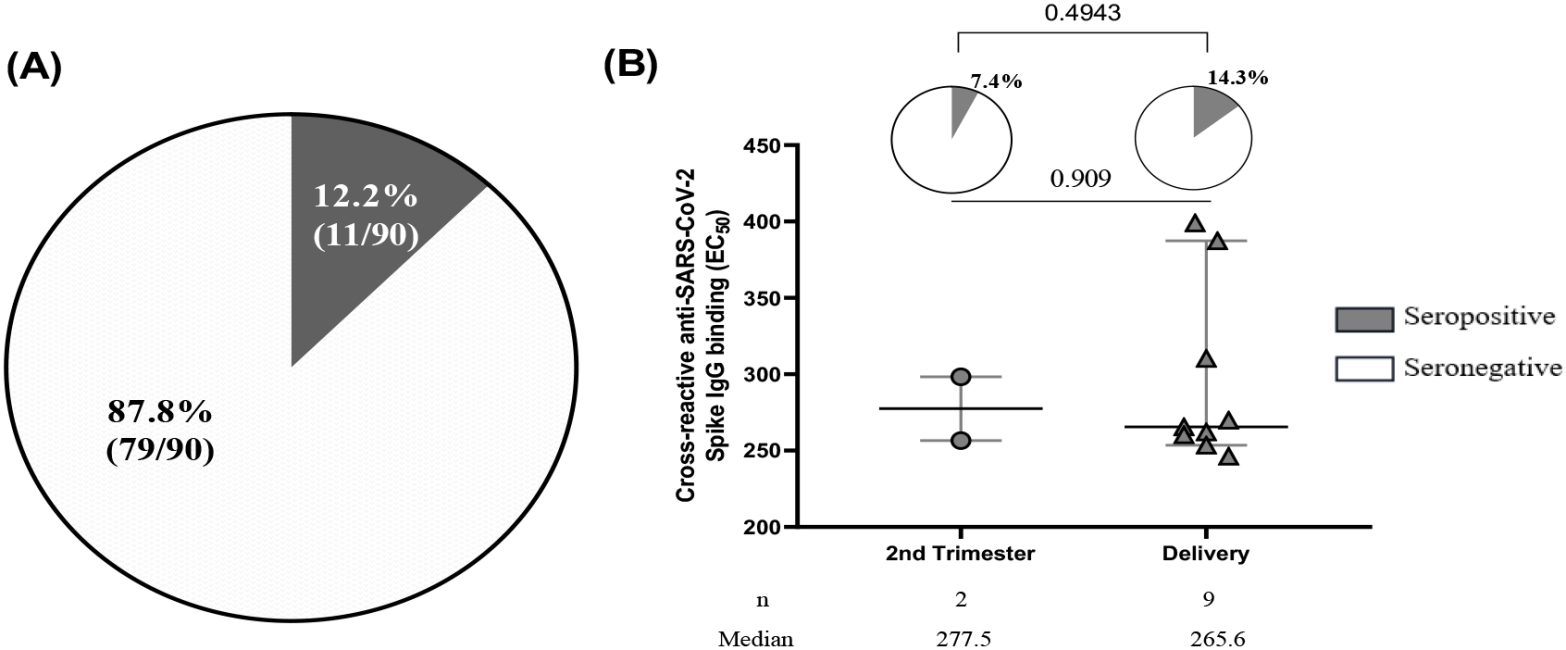
Seroprevalence of cross-reactive anti-SARS-CoV-2 binding antibodies. **(A)** Out of 90 plasma samples taken from pre-COVID-19 pregnant women, which included 24 women who were seropositive for IgM+IgM/IgG as determined by RDT and 66 women who were randomly selected, 11 women were found to be seropositive to cross-reactive anti-SARS-CoV-2 spike IgG binding by ELISA. **(B)** Among the 11 pregnant women seropositive by ELISA, the prevalence of women seropositive at the second trimester was 7.4% compared to 14.3% of women at delivery with p= 0.4943. The half maximal effective concentration (EC50) profiles of cross-reactive anti-SARS-CoV-2 antibodies were illustrated for 2 women during their second trimester and 9 women at the time of delivery with a p value=0.909.

Additionally, the study showed a prevalence of pregnant women seropositive for cross-reactive anti-SARS-CoV-2 antibodies was 12.2% (20/90) (**Figure 3A**) with a prevalence of 14.3% for women at delivery, lower compared to those seropositive in the second trimester (7.4%) although not significant (p=0.4943, p=0.909) (**Figure 3B**).

### No neutralization activity against SARS-CoV-2 was detected among pregnant women in pre-COVID-19 samples

After performing both a full antibody screening and titration for 90 samples using ELISA which enabled us to identify samples that can bind efficiently to the SARS CoV-2 antigens (antibody binding), 12.2% (11/90) of pregnant women met that threshold, however, none of the antibodies in these samples were neutralizing.

### Pre-existing immunity to endemic coronaviruses did not correlate with, nor cross-react, SARS-CoV-2 in pregnant women

**Figure 4:**
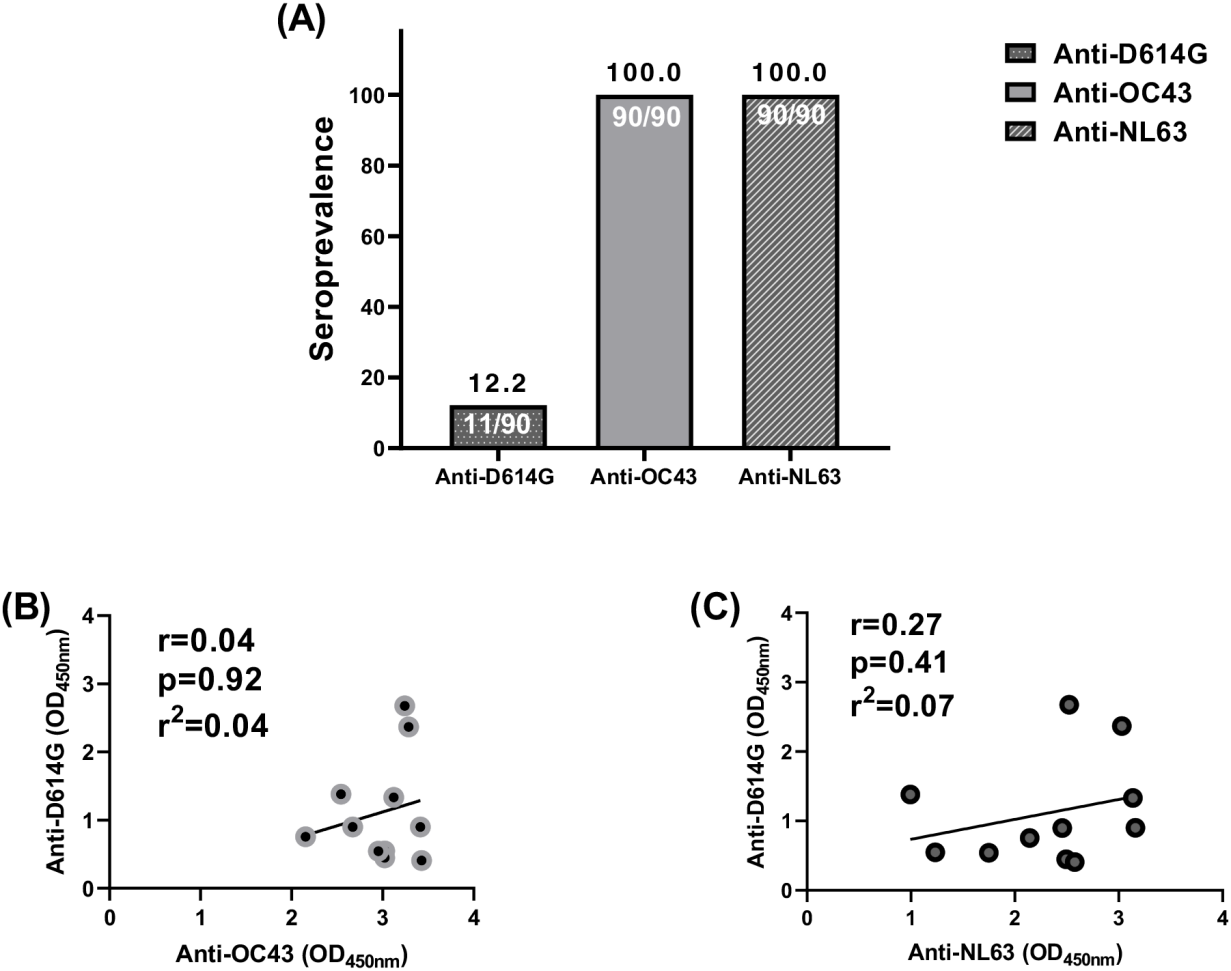
No correlation between cross-reactive anti-SARS-CoV-2 and endemic coronaviruses (OC43 and NL63) antibodies among pregnant women. **(A)** All the 90 plasma samples were seropositive to endemic coronaviruses (OC43 and NL63) antibodies among pregnant women while only 11 were seropositive to cross-reactive anti-SARS-CoV-2 antibody binding. **(B)** The 11 plasma samples did not correlate between cross-reactive anti-SARS-CoV-2 and anti-OC43 antibodies. **(C)** The 11 plasma samples did not correlate between cross-reactive anti-SARS-CoV-2 and anti-NL63 antibodies.

To investigate whether anti-SARS-CoV-2 cross-reactivity in pre-COVID-19 samples correlated with past exposure to other endemic coronaviruses [HCoVs (OC43 and NL63)] in the same samples, pre-COVID-19 plasma samples of pregnant women that cross-reacted with SARS-CoV-2 were tested to determine their anti-HCoVs (anti-OC43 and anti-NL63) responses (**Figure 4**). Among the 90 pregnant women assessed, 22.2% of them had cross-reactive anti-SARS-CoV-2 antibodies directed against the Spike. However, all those women were seropositive to the HCoVs tested in this study (**Figure 4A**). No correlation was found between cross-reactive anti-SARS-CoV-2 (anti-D614G) antibodies and anti-OC43 antibodies (**Figure 4B**) and between (anti-D614G) and anti-NL63 antibodies (**Figure 4C**).

### Anti-D614G antibodies found in women during the pandemic were neutralizing

**Figure 5:**
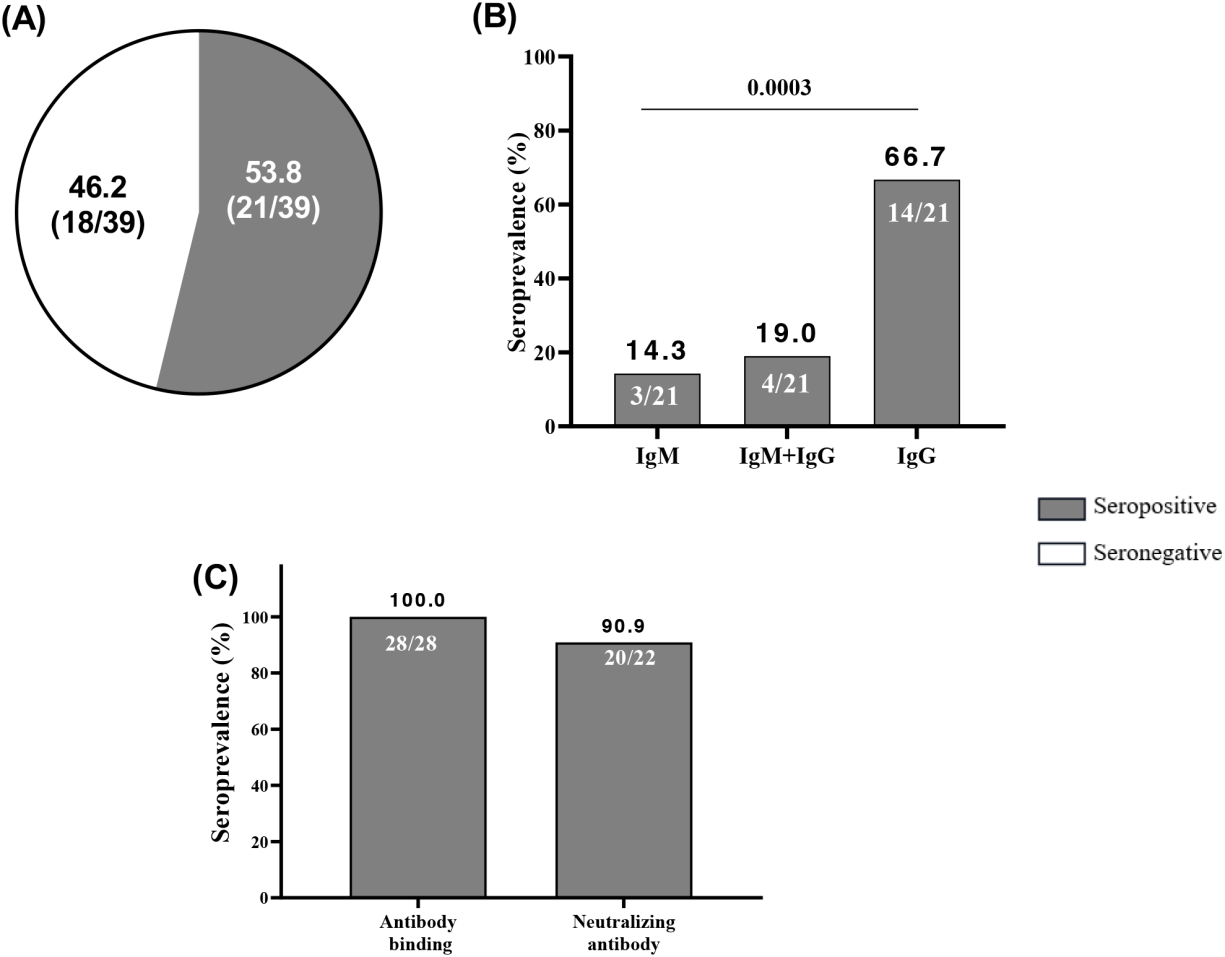
High neutralizing capacity of anti-SARS-CoV-2 antibodies acquired in peri-COVID-19 era. **(A)** Among 39 peri-COVID-19 samples of pregnant women at delivery, 21 were seropositive at anti-SARS-CoV-2 antibodies by RDT. **(B)** Among the 21 peri-COVID-19 seropositive at RDT 3, 4 and 14 were seropositive at IgM, IgM+IgG and IgG respectively. **(C)** Among 28 peri-COVID-19 plasma samples of pregnant women at delivery, all were seropositive at anti-SARS-CoV-2 antibody binding by ELISA and 20/22 had neutralizing antibodies. Immunoglobulin M; IgG, Immunoglobulin G.

To assess cross-neutralization in samples collected during the COVID-19 pandemic as a comparative group, we processed 39 plasma samples of pregnant women at delivery from during the peri-COVID-19 era by RDT. We found that 53.8% (21/39) of women at delivery were seropositive with anti-SARS-CoV-2 antibodies (**Figure 5A**) meanwhile, all these women (n=28) were seropositive to anti-SARS-CoV-2 antibody binding by ELISA (**Figure 5C**). By isotyping the antibodies, more pregnant women expressed higher levels of IgG (p=0.0003) than IgM and IgM+IgG during the pandemic (**Figure 5B**). Overall, 20/22-90.9% of plasma from pregnant women assessed using the spike-pseudotyped lentivirus neutralization assay had neutralizing antibodies.

### Transplacental transfer of anti-SARS-CoV-2 antibodies among pregnant women at delivery

In this study, transplacental transfer was evaluated in pregnant women who showed seropositivity for anti-SARS-CoV-2 antibodies in their peripheral blood. The transfer was assessed by detecting these antibodies both in the maternal peripheral blood and in the corresponding cord blood, which reflects the newborn’s blood. Among the 39 women included in the transplacental transfer analysis of this work, 30 women (76.9%) transferred antibodies by measuring antibodies level in cord blood, while 9 women (23.1%) did not. Specifically, during the pre-COVID-19 period, 4/11 women (13.3%) transferred antibodies and 7/11 women (77.8%) did not. During the peri-COVID-19 period, 26/28 women (86.7%) transferred antibodies and 2/28 women (22.2%) did not. The difference between these groups was statistically significant, with a p<0002. No significant difference (p= 0.6) was observed between the mean maternal age of women who transferred antibodies (28 years old) and women who did not transfer antibodies (28 years old). When looking at the area of residence, 24/31women (77.4%) from peri-urban areas and 6/8 women (75%) from urban areas transferred antibodies. In contrast, 7/31 women (22.6%) from peri-urban areas and 2/8 women (25%) from urban areas did not transfer antibodies. Also, no significant difference was found between primigravida (80%) and multigravida (85.7) and between primiparous (85%) and multiparous (73.3%) who transferred antibodies (p=0.7 and p=0.4 respectively) (**Table 2**).

**Table 2:** Characteristics of the mothers and their newborns.

Characteristics	Women who transferred antibodies	Women who did not transfer antibodies	Total	P value
All women n(%)	**30 (76.9)**	**9 (23.1)**	**39 (100)**	0.0002
Pre-COVID-19	**4 (13.3)**	**7 (77.8)**	**11 (28.2)**	
Peri-COVID-19	**26 (86.7)**	**2 (22.2)**	**28 (71.8)**	
Maternal age, Years old, (mean ± SD)	**28± 5.6**	**28 ± 6.0**	**/**	0.6
Area of residence				
Peri-urban	**24 (77.4)**	**7 (22.6)**	**31**	/
Urban	**6 (75)**	**2 (25)**	**8**	
Gravidity				
Primigravidae	**8 (80)**	**2 (20)**	**10 (26.3)**	
Multigravidae	**24 (85.7)**	**4 (14.3)**	**28 (73.7)**	0.7
Parity				
Primiparus	**17 (85)**	**3 (15)**	**20 (57.1)**	
Multiparus	**11 (73.3)**	**4 (26.7)**	**15 (42.9)**	0.4
Maternal hemoglobin level, g/dL, median [min-max]	**11.3 [7.7-17]**	**11 [9.3-13]**	**/**	0.5
Baby weight, (g), median [min-max]	**3100 [800-3800]**	**3200 [2500-3500]**	**/**	0.4

P values bolded means p<0.05 i.e statistically significant.

**Figure 6:**
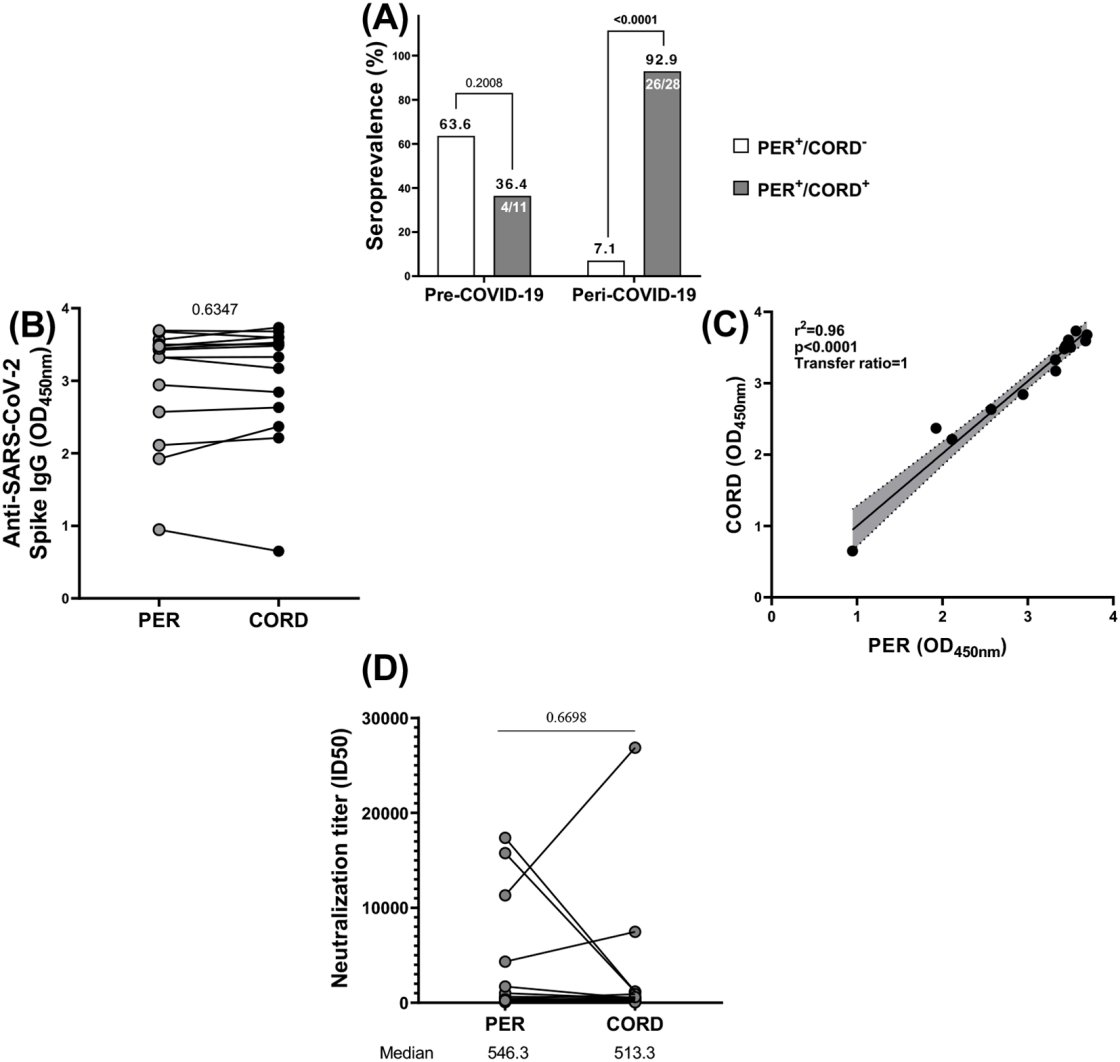
Vertical transmission of anti-SARS-CoV-2 antibodies. **(A)** Among 11 women at delivery seropositive at cross-reactive anti-SARS-CoV-2 antibodies prior COVID-19, 4 transferred antibodies to their newborn and among 28 women at delivery seropositive at anti-SARS-CoV-2 antibodies during COVID-19, 26 transferred antibodies to their newborn. **(B)** All the antibodies generally had the same level of optical density (OD) profile in mothers and newborns. **(C)** All those antibodies were vertically transferred from mothers to newborns. **(D)** the neutralization titer (ID50) of neutralizing anti-SARS-CoV-2 (D614G) antibodies was almost identical in mothers and in newborns. PER, Peripheral blood; CORD, cord blood; + corresponds to seropositive and – corresponds to seronegative.

The efficient transfer of IgG antibodies from seropositive pregnant women to their newborns was also demonstrated in this study (**Figure 6**). Cord blood was used as a surrogacy for newborn blood. Cross-reactive antibodies to SARS CoV-2 were transferred from mother to child prior to and during the pandemic, however, at a lower proportion prior to the pandemic (36.4%) compared to during the pandemic (92.9%). In fact, the transplacental transfer of anti-SAR-CoV-2 antibodies occurred both before and during the COVID-9 pandemic. However, that transfer was significantly represented during the pandemic (p<0.0001) than prior to COVID-19 (p=0.2) (**Figure 6A**). There is no significant difference in the antibody titers between mothers and cord samples that could signify an efficient transplacental transfer of those antibodies from the mother to the fetus (p=0.6347) (**Figure 6B**). There was a strong, statistically significant correlation (r^2^ = 0.96; p<0.0001) between the levels of neutralizing antibodies in mothers and their cords, with the transfer of neutralizing antibodies from mother to cord (ratio=1) (**Figure 6C**).

To evaluate the efficiency of transplacental antibody transfer during the peri-COVID period, we compared the neutralizing capacity of anti-SARS-CoV-2 antibodies between maternal and cord blood samples (**Figure 6D**). Although, there was no significant difference in the neutralizing capacity of anti SAR-CoV-2 antibodies between maternal and their newborns peri-COVID-19 (p=0.6698).

## Discussion

Despite the global drive for COVID-19 immunization, only a few serosurveys and vaccination studies have been conducted in some groups, particularly in low-and-middle-income countries (LMICs) (29). Both vaccine coverage and infection rates may be underestimated as a result of this gap, which makes it challenging to determine the exact level of immunity and infection rates in these groups. Furthermore, pregnant women are not included in most clinical trials and are disproportionately understudied. Therefore, it is crucial to develop strategies to protect both the pregnant woman and her unborn child, as they are equally impacted by the infection which leads to undesirable outcomes (16). To provide more insight into COVID-19-related morbidity and mortality in Cameroon, we i) determined the seroprevalence of cross-reactive anti-SARS-CoV-2 antibodies in pregnant women living in Yaounde, ii) investigated the level of neutralization of these antibodies, iii) assessed the relationship between cross-reactive anti-SARS-CoV-2 (D614G) antibodies and two endemic coronavirus antibodies (anti-OC43 and anti-NL63 antibodies) and iv) show evidence of transplacental transfer of antibodies from mothers to their newborn baby.

In this study, hemoglobin levels in pregnant women at delivery were significantly higher than those in pregnant women in the second trimester. This does not correlate with the literature, which shows that in the third trimester of pregnancy, iron requirements are greater than in the second trimester, thus leading to a hemoglobin peak. However, the high level observed in this study at delivery may be due to either iron supplementation or better health in pregnant women at delivery compared to those in the second trimester (30).

During pre-COVID-19, 16.5% of the pregnant women included in this study were seropositive, with cross-reactive anti-SARS-CoV-2 antibodies by using a lateral flow test and 12.2% by using an in-house ELISA. This is in the same range as previously described (13.5%) in a general population living in Yaounde using the same lateral flow test (9). In addition, the prevalence of pre-existing serological cross-reactivity against SARS-CoV-2 during the pre-COVID-19 era was 19% in Tanzania, 14.1% in Zambia, but 2.4% in USA using the *Immunofluorescence Assay* (IFA) (31). These findings supported the idea that cross-reactivity to SARS-CoV-2 from past exposure to HCoVS could contribute to observations that the global south was less affected by the virus than the global north. In order to test this hypothesis, we investigated whether anti-SARS-CoV-2 cross-reactivity correlated with past exposure to other HCoVs (OC43 and NL63) and we found that, even though all the samples from those pregnant women were seropositive for anti-OC43 and anti-NL63 antibodies, no correlation was found between cross-reactive anti-SARS-CoV-2 antibodies and anti-OC43 and anti-NL63 antibodies in pre-COVID-19 samples from pregnant women.

We found that IgM was significantly (p<0.0001) more represented than IgM+IgG/IgG as was observed in Aissatou’s paper in 2023 who obtained 7.3% (21/288), 7.3% (21/288) and 1.04% (3/288) of individuals seropositive at IgM, IgG and IgM+IgG respectively. We found that the EC_50_ median of cross-reactive anti-SARS-CoV-2 antibodies of pregnant women during the second trimester (277.5) was similar to that at delivery (265.6), (p=0.909). This result is reinforced by others who showed that, during the third trimester, elevated hormone levels led to a reduction in circulating B cells due to their inhibitory effects on lymphopoiesis (32). Additionally, they observed increased cellular migration into tissues, including the placental decidua, which is crucial for maintaining a healthy pregnancy (33). This would suggest that women at delivery might be more vulnerable to SARS-CoV-2 infection than women in their second trimester of pregnancy.

The in-house ELISA assay used to screen samples from 90 pregnant women showed a 12.2% cross-reactive anti-SARS-CoV-2 positivity rate. Our findings are divergent to Souris et al. who explored the presence of pre-pandemic cross-reactive immunity against SARS-CoV-2 among populations in Central and Western Africa (8) and found that, 42.4% of Cameroonians had cross-reactivity against the spike protein of SARS-CoV-2 compared to 24.4% in Congo, 27% in the Democratic Republic of Congo and 20.3% in Senegal (8). The difference in the cross-reactive SARS-CoV-2 positivity rate observed in this study compared to the one found by Souris et al. could be attributed to several key differences concerning the study design and population characteristics. Our cohort is constituted of pregnant women exclusively whose immunological profiles may differ from the general population studied by Souris et al. Also, the sample size in our study is relatively small (n=90) which may limit statistical power and reduce the positivity rate. The methodological differences could also influence the different results obtained between our study and the study of Souris et al. However, we did not find neutralization against the D614G, unlike a study in Vietnam which showed cross-neutralization to the D614G strain in pre-COVID-19 samples (34). Unfortunately, we did not test neutralization against the HCoVs (OC43 and NL63) in the pre-COVID-19 samples and cannot exclude the possibility of cross-neutralization again those coronaviruses. The lower reported cases of COVID-19 in Cameroon among pregnant women might be partially due to cross-reactivity with the spike protein of SARS-CoV-2. However, this cross-reactivity does not provide protection. We also showed that this cross-protection cannot entirely be attributed to prior exposure to HCOVs.

To validate our assays, 39 peri-COVID-19 samples of unvaccinated pregnant women at delivery were processed and we obtained 53.8% compared to 100% (28/28) of pregnant women seropositive to anti-SARS-CoV-2 antibodies by RDT and the in-house ELISA respectively. These results are similar to another study made in Cameroon who showed that 14 months after the beginning of the pandemic, 77% (225/292) unvaccinated pregnant women tested positive (34). It also aligns with the results reported by Ndongo et al., 2022, who documented a rapid increase in SARS-CoV-2 seroprevalence from 18.6% to 75% during the second wave of the pandemic in Yaoundé. This reinforces the evidence of widespread community transmission and supports the relevance of our findings in pregnant women, a population often underrepresented in surveillance efforts. Our study thus contributes to the broader understanding of SARS-CoV-2 exposure in Cameroon and highlights the importance of targeted serological monitoring in vulnerable groups (35). Another study in Tanzania showed a high seroprevalence of 94.0% amongst care workers (36). They conducted a study on the increasing seropositivity of SARS-CoV-2 in Yaoundé, Cameroon which involved repeated cross-sectional serosurveys among adult blood donors in the city and found a significant increase in seroprevalence over time, indicating widespread community transmission of the virus. In our study, approximately 90.9% (20/22) of those women showed neutralizing activity against SARS-CoV-2 which means high exposure confers high protection against the disease. The high prevalence of neutralizing antibodies in the peri-pandemic group could be explained by multiple exposures to SARS-CoV-2, despite no vaccination against SARS-CoV-2. This is in contrast to the pre-pandemic group, where no neutralization was observed. This highlights the importance of a vaccine against COVID-19 in pregnant women and booster doses.

The effectiveness of the transplacental transfer of antibodies from mother to child was also assessed in this study. It showed that the transplacental transfer of antibodies occurred in majority of cases (92.9%) during the peri-COVID era (p<0.0001) compared to pre-COVID-19 era (36.4%) (p=0.2). Our findings concurred with those of Vercoutere et al. who also found high rates of placental transfer of antibodies (81.3%) in their cohort of unvaccinated pregnant women in Brussels during the pandemic (37). This may be due to the exposure of these women to a cocktail of SARS-COV-2 antigen during the pandemic compared to those from pre-COVID-19 era who were not exposed. Although there is no significant difference between the neutralizing activity in mothers and their newborns, there is a slight increase in the level of antibodies in the mother’s blood compared to the level of these antibodies in cord blood. Increased maternal IgG levels were positively associated with those in the umbilical cord. The latter could be due to maternal infection occurring during the early stages of the second and third trimesters of pregnancy (38). The peak production of anti-SARS-CoV-2 antibodies depends on the timing of exposure, infection, or vaccination, occurring 2 to 4 weeks after infection or vaccination in every population (39), and not on gestational age. Thus, if infection occurs during the second trimester, maximum antibody levels may be reached at the end of the second trimester or early in the third trimester of pregnancy. However, transplacental transfer of IgG antibodies may depend on gestational age.

This also explains the high placental transfer ratio (PTR) we obtained in this study which aligns with previous studies that have reported similar trends in PTR variation as pertains to the timing of infection and delivery (40). In contrast, the absence of antibodies observed in newborns of seropositive mothers may be attributed to the infection in the last four weeks of pregnancy or to the possible alteration of the Fc glycosylation of SARS-CoV-2 IgG antibodies, influenced by early inflammatory responses (41).

In summary, we demonstrated in this study that before the pandemic, pregnant women had cross-reactive, anti-SARS-CoV-2 antibodies but those antibodies were not neutralizing in contrast to those of the pandemic period. Additionally, the cross-reactivity of anti-SARS-CoV-2 antibodies was not solely due to pre-existing HCoVs antibodies in this cohort. We showed that efficient transplacental transfer of anti-SARS-CoV-2 IgG directed against the S-protein of D614G from mothers to child occurred. Finally, our study highlights the need to vaccinate pregnant women against COVID-19 as we remain uncertain about the extent to which antibodies generated against the Wuhan strain provide cross-protection against other variants of concern.

### Limitations of this study

Limitations include a relatively small sample size and potential biases in the selection process due to inadequate sample size, which may lead to certain groups being underrepresented. Indeed, before being subjected to ELISA testing, the plasma samples were first screened using the Abbot rapid diagnostic test (RDT), which detects the presence or absence of IgG antibodies. This initial screening served as an indicator of potential seropositivity, which is the primary focus of this study. All these procedures significantly reduced the sample size, to the extent that no samples were seropositive to cross-reactive anti-SARS-CoV-2 antibodies in certain areas as in rural area. Such comparisons using ELISA results would likely introduce bias, given the widely disproportionate number of samples tested by ELISA in certain locations. Another biggest shortcomings of this study was the lack of follow-up that could have helped us track exactly how antibody levels behaved between the second and third trimesters. Targeting the second trimester may reflect maternal seroconversion. This is insufficient to assess transplacental transfer of antibodies, as the peak occurs mainly during the third trimester due to increased expression of the neonatal Fc receptor (FcRn) and placental maturity. The lack of detailed investigation into other HCoVs and SARS-CoV-2 variants, which could contribute to cross-protection observed in pre-pandemic samples also limited our findings. To mitigate these issues, we employed rigorous statistical methods, ensured diverse sampling, and validated our findings with multiple approaches to enhance the reliability and generalizability of our results. We also analyzed peri-pandemic samples to assess long-term immunity and protection levels in mothers and children. Another key limitation of this study is the inability to include vaccinated pregnant which restricted our capacity to assess vaccine-related immune responses throughout pregnancy because current national data on SARS-CoV-2 vaccination in pregnant women remain limited. In this context, our study, although focused on pre-vaccination samples, highlights the critical need for strengthened surveillance systems capable of monitoring antibody responses and vaccine impact in pregnant women.

## Data Availability

The original contributions presented in the study are included in the article/Supplementary Material. Further inquiries can be directed to the corresponding author.
